# Preparation of Red Ginseng Marc-Derived Gintonin and Its Application as a Skin Nutrient

**DOI:** 10.3390/nu15112574

**Published:** 2023-05-31

**Authors:** Rami Lee, Ji-Hun Kim, Hongik Hwang, Hyewhon Rhim, Sung-Hee Hwang, Ik-Hyun Cho, Do-Geun Kim, Hyoung-Chun Kim, Seung-Yeol Nah

**Affiliations:** 1 Ginsentology Research Laboratory and Department of Physiology, College of Veterinary Medicine, Konkuk University, Seoul 05029, Republic of Korea; 2 Department of Life Science, University of Seoul, Seoul 02504, Republic of Korea; 3 Center for Neuroscience, Korea Institute of Science and Technology, Seoul 02792, Republic of Korea; 4 Department of Pharmaceutical Engineering, College of Health Sciences, Sangji University, Wonju 26339, Republic of Korea; 5 Department of Convergence Korean Medical Science, College of Korean Medicine, Kyung Hee University, Seoul 02447, Republic of Korea; 6 Korea Brain Research Institute (KBRI), 61, Cheomdan-ro, Dong-gu, Daegu 41062, Republic of Korea; 7 Neuropsychopharmacology and Toxicology Program, College of Pharmacy, Kangwon National University, Chunchon 24341, Republic of Korea

**Keywords:** Korean red ginseng marc, gintonin, LPA receptors, skin damage, cosmetics

## Abstract

Ginseng is one of the traditional herbal medicines for tonic. Gintonin is a new material derived from white/red ginseng and its lysophosphatidic acids (LPAs) play as a ligand for G protein-coupled LPA receptors. Korean red ginseng marc (KRGM) is a by-product after the KRG processes. We developed a low-cost/high-efficiency method for KRGM gintonin production. We further studied the KRGM gintonin-mediated anti-skin aging effects under UVB exposure using human dermal fibroblasts (HDFs). KRGM gintonin yield is about 8%. KRGM gintonin contains a high amount of LPA C_18:2_, lysophosphatidylcholine (LPC), and phosphatidylcholine (PC), which is similar to white ginseng gintonin. KRGM gintonin induced [Ca^2+^]_i_ transient via LPA1/3 receptors and increased cell viability/proliferation under UVB exposure. The underlying mechanisms of these results are associated with the antioxidant action of KRGM gintonin. KRGM gintonin attenuated UVB-induced cell senescence by inhibiting cellular β-galactosidase overexpression and facilitated wound healing. These results indicate that KRGM can be a novel bioresource of KRGM gintonin, which can be industrially utilized as new material for skin nutrition and/or skin healthcare.

## 1. Introduction

Ginseng is an herbal medicine that is used as a tonic [1]. Ginseng contains various ingredients, including ginsenosides, gintonin, polysaccharides, and other minor components. After harvesting the roots, fresh ginseng is usually further processed into several longer-lasting forms, including white ginseng, Korean red ginseng (KRG), and black ginseng [2]. The processing of fresh ginseng to KRG leads to many changes in the ginseng component [3]. For example, minor ginsenosides are found in KRG that do not exist in fresh ginseng [4,5]. In addition to ginsenosides, it is possible that processing fresh ginseng introduces other ginseng components, although this has not yet been confirmed.

KRG is currently used as a major ginseng product in the Korean ginseng industry for various human applications [6]. There are two ways to process KRG. One way is to conserve the original form of the main root after the removal of any small or thin side roots. The human-shaped main root of KRG is expensive. Additionally, KRG can be extracted by steeping the fresh root in hot water for several days [7,8]. This process is not particularly efficient with 40–50% of KRG remaining in the water after the extraction of the main root, despite KRG being more precious than other herbal medicines. These KRG residues, obtained after hot water extraction, are termed KRG marc (KRGM) [9,10]. The majority of this KRGM is either used as compost and animal feed or discarded, although Korea’s Ministry of Food and Drug Safety (MFDS) admits to using it as a food additive [11]. Currently, the Korean ginseng industry produces a large amount of KRGM (~8000 tons annually), very little of which is upcycled or recycled [12]. This is an important loss given that KRGM still contains useful ginsenosides and ginseng polysaccharides [13,14]. Additionally, KRGM may contain other useful bioactive components, although these have not yet been identified.

Gintonin is a recently discovered glycolipoprotein complex that is a non-saponin component of ginseng [15,16]. The bioactive molecules in gintonin are lysophosphatidic acids (LPAs). LPA C_18:2_ is a major component among LPAs [15]. In vitro and in vivo studies using gintonin stimulate collagen and hyaluronic acid releases and facilitates skin wound healing via G protein coupled LPA1/3 receptors [17,18]. In this study, we examined the presence of KRGM-derived gintonin and found that LPA C_18:2_ in KRGM-derived gintonin (referred to as KRGM gintonin) was high. In addition, we also identified that KRGM gintonin also contains a high amount of lysophosphatidylcholine (LPC) and phosphatidylcholine (PC). Here, we characterized KRGM gintonin in detail and further performed application studies for skin nutrition through a skin aging model under UVB exposure. This report raises a possibility that KRGM, usually used as animal feed or discarded as a waste product, could be utilized for the KRGM gintonin preparation, which might be further used as a skin nutrient for anti-aging as well as a food additive in the future.

## 2. Materials and Methods

### 2.1. Materials Preparation

KRGMs were obtained from local ginseng processors. All other reagents were purchased from Sigma-Aldrich (St. Louis, MO, USA). 1-Linoleoyl-2-hydroxy-sn-glycero-3-phosphate (LPA C_18:2_) was purchased from Echelon (Salt Lake City, UT, USA), and 1-palmitoyl-2-linoleoyl-sn-glycero-3-phosphatidylcholine (PC C_16:0–18:2_ and C_16:0–18:2_) was purchased from Avanti Polar Lipids (Alabaster, AL, USA). Ethanol was purchased from Korea Ethanol Supplies Company (Seoul, Republic of Korea).

### 2.2. Preparation of KRGM Gintonin

**Figure 1:**
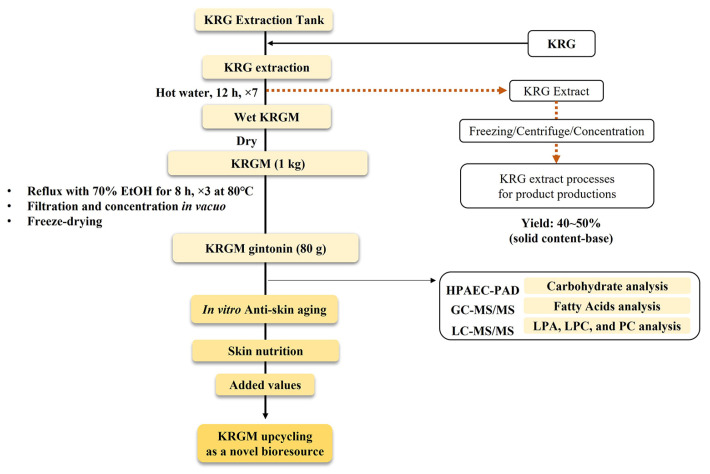
Schematic methods for the preparation of KRGM gintonin. The method uses only edible ethanol and water. KRG extraction first takes place with distilled water for 12 h, 4 to 7 times continuously, to produce KRG extract, which is further processed for KRG product productions, leaving KRGM as a leftover. The KRGM is dried, and then the dry KRGM was refluxed with 70% ethanol for 8 h at 80 °C three times; the air-dried or freeze-dried ethanol extract is about 8% yield of solid content. The resulting powder obtained from the air-dry process was designated KRGM gintonin.

Figure 1 illustrates the method used to obtain KRGM gintonin from KRG. First, KRG was steeped in hot water and 1 kg of KRGM was extracted after several days. This KRGM was then ground into small pieces (>3 mm) and refluxed with 70% ethanol thrice for 8 h at 80 °C. The biological effects of KRGM gintonin were assessed using in vitro experiments in the present study.

### 2.3. Carbohydrate Analysis of KRGM Gintonin

Each sample was pretreated with trifluoroacetic acid (TFA) to give a final concentration of 2 mg/mL. The carbohydrates of the samples were measured according to the HPAEC-PAD system (Dione Co., New York, NY, USA) using a Carbowax PA1 column (eluent, 18 mM NaOH/200 mM NaOH; flow rate, 1.0 mL/min; injection volume, 20 μL) [16].

### 2.4. Fatty Acids Analysis of KRGM Gintonin

A quantitative analysis of the fatty acids was performed using gas chromatography time-of-flight mass spectrometry (GC TOF-MS), after transesterification of the fatty acids to fatty acid methyl esters (FAME). Specifically, a GC TOF-MS analysis was performed using an Agilent 7890B gas chromatograph equipped with a Pegasus 4D (Leco, St. Joseph, MI, USA) time-of-flight mass spectrometer (TOF-MS). Chromatographic separation was achieved using a DB-5MS UI capillary column (30 m × 0.25 µm I.D.; 0.25 µm film thickness) from J&W Scientific (Santa Clara, CA, USA). The GC oven temperature was set at 60 °C for the first 5 min, then increased to 240 °C, in 4 °C increments/min, before being increased, in 10 °C increments, to the final temperature of 310 °C. The temperature of the injector was 280 °C, and the column flow rate was set at 1.0 mL/min with a split ratio of 5:1. The TOF-MS was operated in electron impact (EI) mode at 70 eV electron energy. The mass spectrometry data were acquired in the range of *m*/*z* 45–600, with an acquisition rate of 10 spectra/s.

### 2.5. LPA, LPC, and Phosphatidylcholine Analysis of KRGM Gintonin

The LPA C_18:2_, LPC C_18:2_, and phosphatidylcholine (PC) C_16:0–18:2_/PC C_18:0–18:2_ contents in the KRGM gintonin were determined using liquid chromatography with tandem mass spectrometry LC-MS-MS. The standard markers for LPA, LPC, and PC were identically prepared in high-pressure liquid chromatography (HPLC)-grade methanol, and the solutions were stored at 4 °C. An Agilent series 1100 HPLC instrument (Agilent Technologies, Santa Clara, CA, USA) and an API 2000 LC-MS/MS system (Applied Biosystems, Foster City, CA, USA) were used as previously described [15,19]. The resultant data were given as mean ± relative standard deviation (%) from three different samples of KRGM gintonin.

### 2.6. Intracellular Ca^2+^ Assay

Intracellular Ca^2+^ was measured in a Fura-2 loaded cell using an intracellular ion measurement system (RF-5300PC; Shimadzu Corporation, Kyoto, Japan). Specifically, Fura-2 loaded cells were diluted to a final concentration of 2 × 10^6^ cells/mL and transferred to a polystyrene cuvette (Elkay Ultra–UV), in accordance with other studies [20]. All values are presented as the mean ± relative standard deviation (%) of the samples in the presence or absence of KRGM gintonin. The ratios of the absorbance values measured between 304 nm and 380 nm were converted to [Ca^2+^]_i_ using the formula by Grynkiewicz et al. [21].

### 2.7. Cell Viability Assay

The viability of the HDFs was assessed using a WST–8 (2-(2-methoxy-4-nitrophenyl)-3-(4-nitrophenyl)-5-(2,4-disulfophenyl)-2H-tetrazolium, monosodium salt)-based assay, as described in the manufacturer’s instructions (MONOBIO INC, Seoul, Republic of Korea). First, the cells were seeded at 1 × 10^5^ cell density per well in 96-well plates in complete MEM–α media and exposed to the indicated concentrations of KRGM gintonin (0.1, 0.3, 1, 3, 10, and 30 µg/mL) or LPA (10 µM) for 24 h. Second, the cells were washed twice with 1× DPBS and replaced with a fresh medium with Chromo–CK^TM^ 10 µL for 2 h. Absorbance was measured at 450 nm in a multi-plate reader (SpectraMax ABS Plus, Molecular Devices, San Jose, CA, USA).

### 2.8. Analysis of ABTS^+^ Radical Scavenging Activity (H_2_O_2_-Specific Test)

The ABTS radical decolorization assay of the KRGM gintonin was performed, with minor modification, as per the procedures of Arnao et al. [22]. Briefly, ABTS cation radicals were produced by mixing 14 mM ABTS and 4.9 mM potassium persulfate in distilled water. The two solutions were mixed in equal quantities, placed in the dark for 16 h, and further diluted with ethanol to adjust the OD value to 0.700 ± 0.02 at 734 nm. After centrifugation, the supernatants were diluted and filtered, 1.56, 3.12, 6.25, and 10 mg/mL, respectively. Tests were performed in triplicate and the results were calculated as described by Prior et al. [23].

### 2.9. Nitric Oxide Analysis

The cells were seeded in 96 wells at a density of 1 × 10^5^ cells/well and either treated with KRGM gintonin (0.1, 0.3, 1, 3, 10, and 30 μg/mL) or LPA (10 μM), as a control, for 24 h. Cell supernatants were collected for further analysis. The modified Griess reagent (Sigma–Aldrich, G4410, St. Louis, MO, USA) was added to the collected supernatant at room temperature for 15 min. The optical density (O.D.) at 540 nm was then measured using a multi-plate reader (SpectraMax ABS plus, Molecular Devices, San Jose, CA, USA).

### 2.10. Intracellular Reactive Oxygen Species Analysis

CM-H2DCFDA, which acts as a non-fluorescent reactive oxygen species (ROS) indicator (Invitrogen, Carlsbad, CA, USA), was used to analyze the intracellular reactive oxygen species (ROS) levels. The HDF was seeded at 2 × 10^5^ cells/mL in an 8-well chamber slide (Nunc™ Lab-Tek™ II Chamber Slide™ System, Thermo Fisher Scientific, Waltham, MA, USA), and left overnight. The cells were then washed twice with PBS, irradiated with UVB at 50 mJ/cm^2^, and treated with either KRGM gintonin at 3 μg/mL or 10 μM of LPA. The UVB-irradiated cells were used as a positive control, whereas untreated cells were used as a negative control. After 24 h, the medium was removed and replaced with 20 μM CM-H2DCFDA in serum-free MEM-α for 30 min at 37 °C in the dark. After washing three times with PBS, the cells were fixed in 4% paraformaldehyde in PBS for 20 min at room temperature. The cells were stained for nuclei and mounted with Vectashield Mounting Media (Vector Laboratories, Burlingame, CA, USA) containing 4′,6-diamidino-2-phenylindole (DAPI). Images were captured using an Axio200 inverted fluorescence microscope (Carl Zeiss, Oberkochen, Baden-Württemberg, Germany) equipped with a green fluorescence filter.

### 2.11. Intracellular Reactive Oxygen Species Analysis

Senescence-associated beta-galactosidase (SA-β-Gal) staining was performed, according to the manufacturer’s instructions, using an SA-β-Gal staining kit (9860; Cell Signaling Technology, Danvers, MA, USA). Senescent cells were identified as blue–stained under a light microscope (Zeiss Axio Imager bright-field microscopy, Zeiss, Jena, Germany). The cells were seeded at a density of 3 × 10^5^ cells/well in a 12-well plate and grown overnight. The complete growth medium was then removed, and the cells were washed twice with PBS. KRGM gintonin was then added and the medium was irradiated with UVB at 50 mJ/cm^2^ power strength, before being left for another 24 h. After the growth media were removed, the cells were rinsed twice with 1× DPBS, stained for 15 min at RT with a kit for detection of senescent of cells fixed with 1× Fixative Solution, and then washed again. A ꞵ-Galactosidase staining solution was then added, and the cells were incubated at 37 °C overnight. When the blue color was fully developed, the percentage of blue SA-β-gal-positive cells was counted under a microscope. The SA-β-gal-positive cells stained blue-green and were scored using a Zeiss Axio Imager bright-field microscope. Results were obtained for at least three independent experiments. More than 500 cells were counted for each experimental condition.

### 2.12. Scratch Wound Healing Assay

An in vitro wound-healing assay was performed as follows: The cells were seeded into 24-well plates at a density of 2.5 × 10^5^ cells/well and incubated at 37 °C in a CO_2_ incubator for 24 h. These cells were incubated in a serum-free medium at 37 °C for 2 h. The cell layers were scratch-wounded using a 200 μL pipette tip and then washed in a serum-free medium before being incubated with different concentrations of KRGM gintonin (0.3, 1, 3, 10, 30 µg/mL) or LPA (10 µM) at 37 °C for 24 h. Images were captured using an inverted fluorescence microscope (AxioVert200; Carl Zeiss, Oberkochen, Germany) at a magnification of ×100 and analyzed using an image analysis software (AxioVision Rel. 4.8, White Plains, NY, USA). The wound-closure ratio was calculated as the recovered area at 24 h divided by the initial wound area at 0 h. This ratio was then compared to the untreated control group to give the percentage of wound closure.

## 3. Results

### 3.1. Preparation of KRGM Gintonin from KRGM

The extraction of KRG using hot water is a common practice, leaving behind KRGM as part of the residue (Figure 1). We then obtained an ethanol extract of KRGM from this residue (Figure 1), gaining approximately 80 g of KRGM/kg of KRG. This yield, 8% KRGM, is relatively high (Figure 1), and the procedure itself could easily be applied to the mass production of KRGM-derived gintonin from KRGM. Moreover, the analysis showed that the LPA C_18:2_ content in KRGM gintonin (Table 1), which is a functional indicator or marker of gintonin, was as high as that of the gintonin-enriched fraction typically obtained from white ginseng [19].

### 3.2. Quantitation of LPA C_18:2_, LPC C_18:2_, and Phosphatidylcholine (PC C_16:0–18:2_ and C_16:0–18:2_) Free Fatty Acids in KRGM Gintonin

The LPA C_18:2_, lysophosphatidylcholine (LPC C_18:2_), and phosphatidylcholines (PC C_16:0–18:2_ and C_16:0–18:2_) contents in the KRGM gintonin were analyzed using LC-MS/MS [19]. KRGM gintonin contained relatively high amounts of LPA C_18:2_, LPC C_18:2_, and PC C_16:0–18:2_/PC C_18:2–18:2_ (Table 1). The LPA C_18:2_, LPC C_18:2_, PC C_16:0–18:2_, and PC C_18:2–18:2_ contents were 0.27%, 0.99%, 1.38%, and 1.48%, respectively. The order of contents was PC C_18:2–18:2_ > PC C_16:0–18:2_ > LPC C_18:2_ > LPA C_18:2_. Interestingly, we could not quantify PA and other minor phospholipids in the KRGM gintonin. These results show that KRGM gintonin contains a high amount of LPC and PC compared to the previous gintonin-enriched fraction prepared from white ginseng.

### 3.3. [Ca^2+^]_i_ Transient Induction by KRGM Gintonin

**Figure 2:**
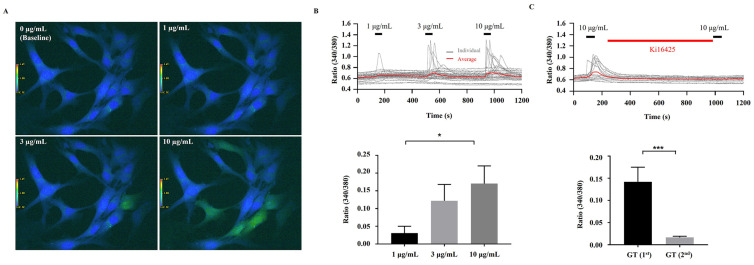
Effects of KRGM gintonin (GT) on [Ca^2+^]_i_ transient in HDFs. (**A**) Comparison of [Ca^2+^]_i_ transient in human dermal fibroblasts (HDFs) by KRGM gintonin. Treatment concentrations of KRGM gintonin are 0, 1, 3, or 10 μg/mL. (**B**) Histograms of [Ca^2+^]_i_ transient in HDF cells induced by KRGM gintonin at three representative concentrations (0, 1, 3, and 10 μg/mL). [Ca^2+^]_i_ transient was elicited in a dose-dependent manner. The strongest [Ca^2+^]_i_ transient was exhibited at 10 μg/mL. (**C**) Inhibitory effect of [Ca^2+^]_i_ transient in HDFs induced by KRGM gintonin by Ki16425 (10 μM) after KRGM 10 μg/mL treatment. * *p* < 0.05, compared with baseline (0 μg/mL); *** *p* < 0.05, compared with 10 μg/mL treatment group. Statistical analysis was conducted by *t*-test. Each graph shows the mean ± SEM of three independent experiments.

Since KRGM gintonin contains LPA C_18:2_, and these LPAs induce [Ca^2+^]_i_ transients in cells that express endogenous LPA receptors, we also examined the effects of KRGM gintonin on [Ca^2+^]_i_ transients using human dermal fibroblasts that endogenously express LPA receptors [17]. Cells treated with KRGM gintonin showed a dose-dependent response induction of the [Ca^2+^]_i_ transient, but this response was blocked by Ki16425, an LPA1/3 receptor antagonist (Figure 2). These results show that LPA in KRGM gintonin is responsible for the [Ca^2+^]_i_ transient induction via LPA1/3 receptors (Figure 2).

### 3.4. Effects of KRGM Gintonin on the Viability of HDFs Exposed to UVB

**Figure 3:**
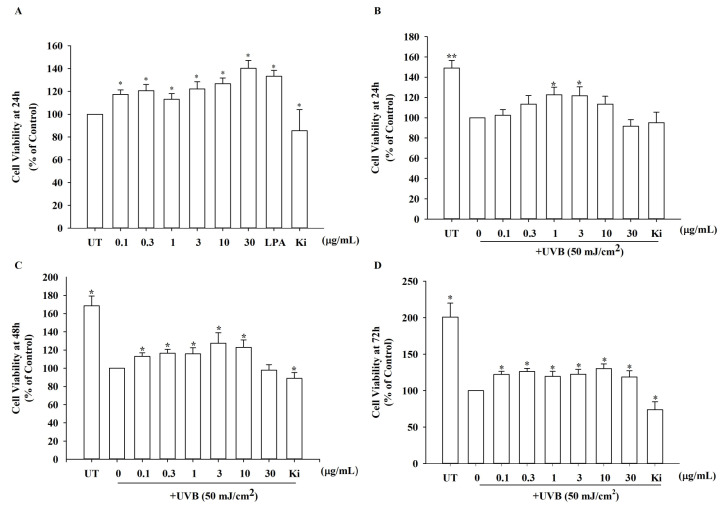
Cell cytotoxicity and cell protection assay by KRGM gintonin in HDFs. HDFs were seeded in a 96-well plate at 2 × 10^4^ cells/well density for assay. (**A**) Cell viability assay using KRGM gintonin (GT) at 24 h. Each treatment group with indicated doses exhibited a significant increase. * *p* < 0.05, compared with the untreated group. (**B**–**D**) Cell viability assay using KRGM gintonin at 24 h, 48 h, and 72 h under ultraviolet (UV) damage at a power of 50 mJ/cm^2^. HDFs were treated with KRGM gintonin. (**B**) KRGM gintonin treatment increased cell viability at a concentration of 1 and 3 μg/mL under UVB exposure at 24 h. (**C**) KRGM gintonin treatment increased cell viability in a dose-dependent manner from 0.1 to 10 μg/mL under UVB exposure at 48 h. (**D**) KRGM gintonin treatment increased cell viability at every dose under UVB exposure at 72 h. * *p* < 0.05, compared with the control group. ** *p* < 0.01, compared with untreated group. Each graph shows the mean ± SEM of three independent experiments. UT; untreated control group, Ki; Ki16425 10 μM treatment group.

Our investigation of how KRGM gintonin influences the viability of HDF cells revealed that KRGM gintonin did not exhibit cytotoxicity at any of the doses tested, but instead stimulated cell proliferation in a dose-dependent manner (Figure 3A). Next, we examined the protective effects of KRGM gintonin on HDFs exposed to UVB. As shown in Figure 3B–D, cell viability decreased after UVB exposure. However, KRGM gintonin inhibited cell death upon UVB exposure in a dose-dependent manner (Figure 3B–D). However, co-treatment of Ki16425 with KRGM gintonin under UV exposure attenuated the KRGM gintonin-mediated protective effects against UV damage (Figure 3B–D).

### 3.5. Effects of KRGM Gintonin on ABTS^+^ Radical, NO, and Reactive Oxygen Species (ROS) Production

**Figure 4:**
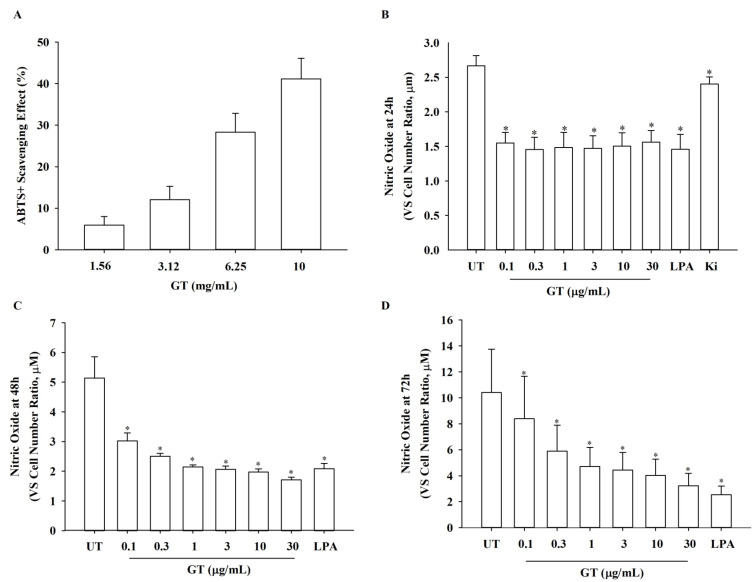
Antioxidant effect of KRGM gintonin (GT) and inhibition of NO production by KRGM gintonin (GT). (**A**) KRGM gintonin effects on ABTS^+^ radical scavenging activity. ABTS^+^ radical scavenging activity was measured using KRGM gintonin. KRGM gintonin showed the strongest radical scavenging activity in the 10 mg/mL group. The ABTS^+^ radical scavenging effect of KRGM gintonin increased in a dose-dependent manner. Each bar indicates the mean ± S.D. (*n* = 4). Each graph shows the mean ± SD of four independent experiments. (**B**–**D**) HDFs were seeded in a 96-well plate at a 1 × 10^4^ cells/well density for assay. HDFs were treated with KRGM gintonin. (**B**) Every dose of KRGM gintonin (from 0.1 to 10 μg/mL) showed a significant decrease in NO production at 24 h. * *p* < 0.05, compared with the untreated control group). (**C**) Every dose of KRGM gintonin (from 0.1 to 10 μg/mL) showed a significant decrease at 48 h, showing the most decrease in NO production at 30 μg/mL. * *p* < 0.05, compared with the untreated control group). (**D**) Every dose of KRGM gintonin (from 0.1 to 10 μg/mL) showed a significant decrease at 72 h, showing the most decrease in NO production at 30 μg/mL. * *p* < 0.05, compared with the untreated control group). Each NO produced was shown as the concentration of NO vs. cell number ratio (μM). Each graph shows the mean ± SD of three independent experiments. UT; untreated control group, LPA; LPA treatment group (10 μM), GT; KRGM gintonin treated group.

**Figure 5:**
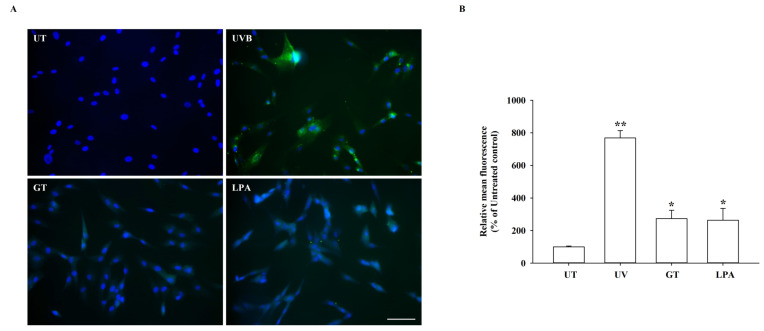
KRGM gintonin (GT) effect on reactive oxygen species (ROS) production in UVB-damaged HDFs. HDFs were seeded in an 8-well chamber at a 1 × 10^5^ cells/well density for assay. (**A**) HDFs treated with/or without KRGM gintonin (10 μg/mL) or LPA (10 μM). (**B**) Protective effect of KRGM gintonin against UVB damage, which generates ROS. UVB irradiation caused HDFs damage, co-treatment of KRGM gintonin protected cells against UVB irradiation, and LPA as a positive control showed a cell-protective effect. * *p* < 0.05, compared with non-treatment of UVB. ** *p* < 0.001, compared with non-treatment of UVB. Each graph shows the mean ± SD of three independent experiments. UT; untreated control, LPA; LPA treated control (10 μM), UV; UVB 50 mJ/cm^2^, GT; KRGM gintonin treated group.

To test whether KRGM gintonin has free radical scavenging properties, we assessed the degree of ABTS^+^ radical removal using KRGM gintonin. This revealed that KRGM gintonin eradicates ABTS^+^ radicals in a dose-dependent manner, with the highest effect observed at 10 mg/mL (Figure 4A). We subsequently examined the inhibition of nitric oxide (NO) and reactive oxygen species (ROS) production after KRGM gintonin treatment. As shown in Figure 4 and Figure 5, treatment of HDFs with KRGM gintonin attenuated UVB-induced NO and ROS production in a dose- and time-dependent manner (Figure 4B–D and Figure 5).

### 3.6. Effects of KRGM Gintonin on Cell Aging and In Vitro Wound Healing under UVB Exposure

**Figure 6:**
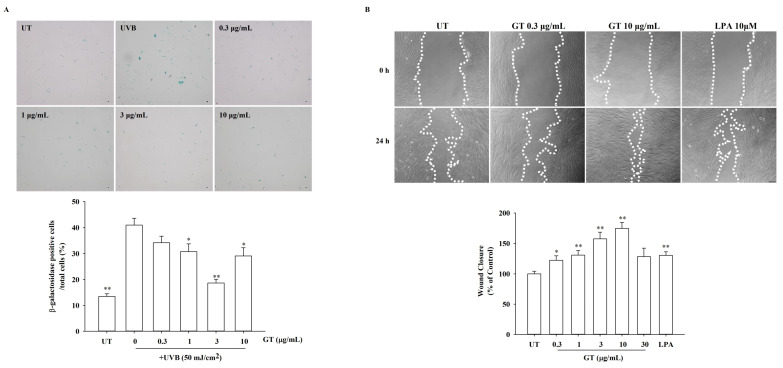
Effects of KRGM gintonin (GT) on cellular senescence and on scratch wound healing. (**A**) SA-β–galactosidase assay in HDFs upon KRGM gintonin treatment. Untreated control, UVB 50 mJ/cm^2^ treated, UVB 50 mJ/cm^2^ + KRGM gintonin (0.3 μg/mL), UVB 50 mJ/cm^2^ + KRGM gintonin (1 μg/mL), UVB 50 mJ/cm^2^ + KRGM gintonin (3 μg/mL), and UVB 50 mJ/cm^2^ + KRGM gintonin (10 μg/mL). The histograms are depicted as a percentage of the β–galactosidase positive cells among total cells. β–galactosidase positive cells are relatively low in the UVB-untreated group but high under UVB exposure. The restoring effects from UVB damage were observed at 1–3 μg/mL of KRGM gintonin. * *p* < 0.05, compared with the control group. Each graph shows the mean ± SEM of three independent experiments. ** *p* < 0.001, compared with the control group. Each graph shows the mean ± SEM of three independent experiments. (**B**) Effects of gintonin on scratch wound healing of HDFs. After the serum was removed for 24 h, cells were scratched with a micropipette tip and then incubated with a serum-free medium for another 24 h containing KRGM gintonin (0.3–30 μg/mL) and lysophosphatidic acid (LPA, 10 μM) as a positive reference agent. Images were obtained by taking photos at 0 h and 24 h post-treatment with KRGM gintonin or LPA. Representative images of control cells, cells treated with KRGM gintonin (0.3 and 10 μg/mL), and LPA (10 μM). The scale bar is equivalent to 200 μm. LPA was used as reference material for a positive control. Wound closing response of untreated cells (UT) was calculated as 100%. Data are presented as the means ± S.E.M. (*n* = 9); * *p* < 0.05 vs. control, ** *p* < 0.01 vs. control. UT; untreated control, LPA; LPA treated group (10 μM), GT; KRGM gintonin treated group.

We also evaluated the effect of KRGM gintonin on cell aging using HDFs. As a cell aging marker, we examined changes in β-galactosidase expression in the absence or presence of KRGM gintonin and UVB exposure. As shown in Figure 6A, HDFs exposed to UVB alone showed an increased β-galactosidase expression compared to normal HDFs (i.e., those not exposed to UVB). However, when HDFs were exposed to UVB in the presence of KRGM gintonin, these HDFs showed a decreased β-galactosidase expression. Interestingly, the KRGM gintonin-induced attenuation of the β-galactosidase expression was highest at 3 μg/mL. Higher concentrations of KRGM gintonin (10 μg/mL) were less effective than 3 μg/mL for attenuating β-galactosidase expression. We subsequently examined the effects of KRGM gintonin on the in vitro wound healing of HDFs. As shown in Figure 6B, KRGM gintonin increased in vitro wound healing, in a dose-dependent manner. As a positive control, we used LPA C_18:2_ and examined its effect on in vitro wound healing. LPA also increased in vitro wound healing (Figure 6B). Similarly, the KRGM gintonin-induced wound healing effect was highest at l0 μg/mL rather than 30 μg/mL, indicating that the optimum concentration of KRGM gintonin might be different depending on tests. Taken together, these results indicate that KRGM gintonin has both anti-aging and wound-healing properties.

## 4. Discussion

Panax ginseng is one of the precious herbal medicines since ginseng is usually harvested after 4–6 years of cultivation. In addition, the majority of the fresh ginseng in Korea is steamed for the preparation of KRG. KRG is then subjected to hot water extraction for KRG products with yields between 40–50% KRG (Figure 1). The remaining 50–60% of the products of the hot water extraction are usually left as KRGM. Moreover, many red ginseng companies in Korea produce very large amounts of KRGM every year, up to 8000 tons. However, approximately 20% of this is used either to feed farm animals and pets or to use as compost for crops, without upcycling to create added value from KRGM (Figure 1). The remaining 80% of KRGM is discarded as waste, which might not only charge the waste disposal costs of KRGM but also cause environmental problems. Moreover, KRGM was not well utilized in the Korean ginseng industry, despite the MFDS allowing the use of KRGM as a food additive since there is an old concept that KRGM is a kind of leftover or waste. Therefore, it is important to understand how KRGM can be further utilized for upcycling. Here, we developed a novel method for the KRGM gintonin preparation with very low-cost/high efficiency, in which the way for maximum utilization of KRGM upcycling is as a skin nutrient.

To our knowledge, this is the first attempt to determine the amounts of LPA C_18:2_, LPC C_18:2_, and PC C_16:0–18:2_/PC C_18:2–18:2_ in KRGM gintonin. In our previous study, we showed that the gintonin-enriched fraction obtained from white ginseng contained approximately 0.2% LPA C_18:2_ and 1% phosphatidic acid (PA) C_16:0–18:2_, respectively [19]. Importantly, KRGM gintonin contains as high an amount of LPA C_18:2_ as that of a gintonin-enriched fraction of white ginseng, despite KRGM being a leftover of KRG extraction (Table 1) [13]. In addition, we found that KRGM gintonin contains more LPC and PC than that of white ginseng (Table 1). Additionally, KRGM contains a high amount of free fatty acids (>5%), which is in line with previous studies [19]. These fatty acids comprised, from most to least, linoleic acid, palmitic acid, and oleic acid.

Despite these positive findings, it will be questioned how a simple ethanol extraction of KRGM produces KRGM gintonin with such a high amount of LPA, LPC, PC, and fatty acids. It is well known that the compositions of ginsenosides undergo changes when fresh ginseng is steamed under high temperatures and pressures to prepare KRG. For example, ginsenoside Rg3 is rare in fresh ginseng and white ginseng but increases several-fold in KRG. In contrast, ginsenoside Rb1, which is abundant in fresh ginseng, decreases in KRG. These results suggest that the steaming of fresh ginseng removes the carbohydrate backbone of ginsenoside Rb1 and other carbohydrate-rich ginsenosides, thus causing their conversion into ginsenoside Rg3 [8]. Processing fresh ginseng into KRG might also cause changes to the composition of lipids, including phospholipids. In addition, hot water extraction tends to concentrate those KRG extracts that are water-soluble, including carbohydrates, proteins, and some ginsenosides. Consequently, water-insoluble components, such as fatty acids, lipids, and phospholipids, might remain after hot water extraction in KRGM (Table 2). When dry KRGM was processed with a high concentration of ethanol at a high temperature, the water-insoluble lipids, including fatty acids, LPA, LPC, PC, and additional ginsenosides, could be extracted (Table 1, Table 2 and Table 3).

In addition, high pressure, high temperature, and moisture/H_2_O during steaming and/or extraction may affect phospholipids, causing a loss of fatty acids attached to the glycerol backbone to produce lysophospholipids. This scenario is likely given the inherent instability of phospholipids and their tendency to decompose into lysophospholipids by removing one fatty acid molecule under unfavorable conditions, such as high temperatures and pressures. As shown in Table 1, KRGM gintonin, derived via the ethanol-based extraction of KRGM, contains LPC, which is derived from PC following the removal of one fatty acid molecule. Sequential removal of the head polar choline group of LPC results in LPA (Supplementary Materials Figure S1). Therefore, the major source of KRGM gintonin LPA may be LPC and PC. Thus, the PC could be converted into LPC and then the LPC into LPA, which is a major biologically active ingredient for targeting G protein-coupled LPA receptors in mammalian systems. These results collectively suggest that the transformation of PC, LPC, and other minor phospholipids into LPA can occur during the steaming of fresh ginseng into KRG, as observed in the ginsenoside conversions.

We also examined the physiological functions of KRGM. In Figure 2, the [Ca^2+^]_i_ transient induction by KRGM gintonin was confirmed through LPA1/3 receptors. In addition, these results are consistent with those of our previous studies on [Ca^2+^]_i_ transients induced by gintonin but not ginsenosides [17]. Thus, KRGM gintonin LPA C_18:2_ causes the same physiological induction of [Ca^2+^]_i_ transients as gintonin derived from white ginseng. This shows that the calcium-inducing effect is unique to gintonin [15]. Then, cells were exposed to 50 mJ/cm^2^ of UVB, a type of ROS generator that induces cell damage and causes cells to undergo aging [24]. These results indicate that KRGM gintonin-mediated protection against UVB exposure is achieved via the LPA1/3 receptors. These results are consistent with previous reports that LPA receptor activation is closely associated with cell proliferation and survival [18]. Additionally, previous reports show that gintonin can function as an antioxidant [25]. KRGM gintonin-induced antioxidant action may contribute to the inhibition of NO and ROS formation under UVB exposure.

The skin is the largest human organ. One of the roles of the skin, since it covers the entire body, is to provide protection from external threats, such as UV radiation. UV radiation has many deleterious effects on skin cells [26,27]. For example, UV penetrates the skin layer and induces or causes premature skin aging via cell DNA damage, oxidative stress, and cell apoptosis [26,27]. The use of UV protection greatly reduces this deterioration, and so, prevents the appearance of premature aging. In this study, we found that KRGM gintonin was not cytotoxic. KRGM gintonin showed antioxidant effects and protected skin cells from UVB-induced cell damage by inhibiting NO and ROS production. In addition, KRGM gintonin attenuated the UVB-induced acceleration of cell aging and facilitated wound healing. Interestingly, cosmetics containing synthetic LPAs are currently available for skin regeneration. Thus, KRGM gintonin could be a potential candidate for anti-aging skin nutrients, since KRGM gintonin contains a high amount of natural LPA and can be easily produced from KRGM at a low cost with high efficiency.

## 5. Conclusions

Our study provides evidence that KRGM contains high amounts of gintonin LPA and its precursors LPC and PC. Additionally, it suggests an application for KRGM gintonin LPA in skin-related healthcare products. Here, KRGM gintonin could protect, via LPA receptor activation, the skin from UV damage. We also show that KRGM, which has typically been a waste product, contains a valuable source of gintonin LPA. Thus, KRGM is a novel bioresource that can be fully utilized through the preparation of KRGM gintonin. Finally, KRGM gintonin could be highly valuable for skin nutrients and/or skin healthcare, since gintonin also enhances hyaluronic acid and collagen release and facilitates skin wound healing [17,18].

## Figures and Tables

**Table 1:** The amounts of LPA, LPC, and PC in KRGM gintonin (*n* = 5).

	LPA C_18:2_ (μg/mg)	LPC C_18:2_ (μg/mg)	PC C_16:0–18:2_ (μg/mg)	PC C_18:2–18:2_ (μg/mg)
KRGM	2.7 ± 0.41	9.87 ± 1.58	13.8 ± 2.52	14.8 ± 1.59

**Table 2:** Compositions and amounts of fatty acids in KRGM gintonin (μg/mg) (*n* = 4).

Fatty Acids	Concentration (μg/mg)
Pentadecanoic acid (C_15:0_)	1.17
Hexadecenoic acid (C_16:1_)	3.16
Palmitic acid (C_16:0_)	77.28
Heptadecanoic acid (C_17:0_)	1.75
Linoleic acid (C_18:2n6c_)	500.73
Elaidic acid (C_18:1n9t_)	11.62
Oleic acid (C_18:1n9c_)	16.15
Stearic acid (C_18:0_)	2.85
11,14-Eicosadienoic acid (C_20:2_)	1.88
Arachidic acid (C_20:4n6_)	0.45
cis 13,16-Docosedienoic acid (C_22:2_)	0.51
Docosanoic acid (C_22:0_)	0.88

Data are presented as μg/mg. The detailed methods for lipid compositions of KRGM are described in Materials and Methods. (Hexanoic acid (C_6:0_); 0.05%, Octanoic acid (C_8:0_); 0.27%, Dodecanoic acid (C_12:0_); 0.12%, Myristoleic acid (C_14:0_); 0.20%, cis-10-pentadecenoic acid (C_15:1_); 0.12%, cis-10-heptadecenoic acid (C_17:1_); 0.23%, Linolenic acid (C_18:3n6_); 0.09%).

**Table 3:** Carbohydrate compositions and amounts in KRGM gintonin (*n* = 4).

Name	Amount (mg/mg)
Fucose	0.012
Rhamnose	0.008
Arabinose	0.058
Galactose	0.016
Glucose	0.335
Xylose	0.009
Fructose	0.008
Total	0.446

Data are presented as mg/mg. The detailed methods for polysaccharide compositions of KRGM are described in Materials and Methods.

## Data Availability

The data from this work will be made available upon request.

## Supplementary Materials

The following supporting information can be downloaded at: https://www.mdpi.com/article/10.3390/nu15112574/s1, Figure S1: A possible process for the lysophosphatidic acids (LPAs) formation from phospholipids such as phosphatidic acid (PA) or phosphatidylcholine (PC) in KRGM gintonin.
