# Supplementary material for: Preparation of Red Ginseng Marc-Derived Gintonin and Its Application as a Skin Nutrient
Source: Nutrients. 2023 May 31;15(11):2574. doi: 10.3390/nu15112574 (PMC10255325; doi:10.3390/nu15112574)
Supplement: Supplementary file 1 [file nutrients-15-02574-s001.zip › nutrients-2419188-supplementary.pdf]

Nutrients

Supplementary Material

Preparation of red ginseng marc-derived gintonin and its application as a skin nutrient

Rami Lee [1], Ji-Hun Kim [1], Hongik Hwang [2], Hyewhon Rhim [3], Sung-Hee Hwang [4], Ik-Hyun Cho [5], Do-Geun Kim [6], Hyoung-Chun Kim [7] and Seung-Yeol Nah [1,*]

[1] Ginsentology Research Laboratory and Department of Physiology, College of Veterinary Medicine, Konkuk University, Seoul 05029, Republic of Korea; rmlee12@konkuk.ac.kr (R.L.); bioskjh@naver.com (J.-H.K.)
[2] Department of Life Science, University of Seoul, Seoul 02504, Republic of Korea; hongik@uos.ac.kr
[3] Center for Neuroscience, Korea Institute of Science and Technology, Seoul 02792, Republic of Korea; hrhim@kist.re.kr
[4] Department of Pharmaceutical Engineering, College of Health Sciences, Sangji University, Wonju 26339, Republic of Korea; sunghhwang@sangji.ac.kr
[5] Department of Convergence Korean Medical Science, College of Korean Medicine, Kyung Hee University, Seoul, 02447, Republic of Korea; ihcho@khu.ac.kr
[6] Korea Brain Research Institute (KBRI), 61, Cheomdan-ro, Dong-gu, Daegu, 41062, Republic of Korea; kimvet0911@kbri.re.kr
[7] Neuropsychopharmacology and Toxicology Program, College of Pharmacy, Kangwon National University, Chunchon 24341, Republic of Korea; kimhc@kangwon.ac.kr
*Correspondence: synah@konkuk.ac.kr; Tel.: +82-2-450-4154

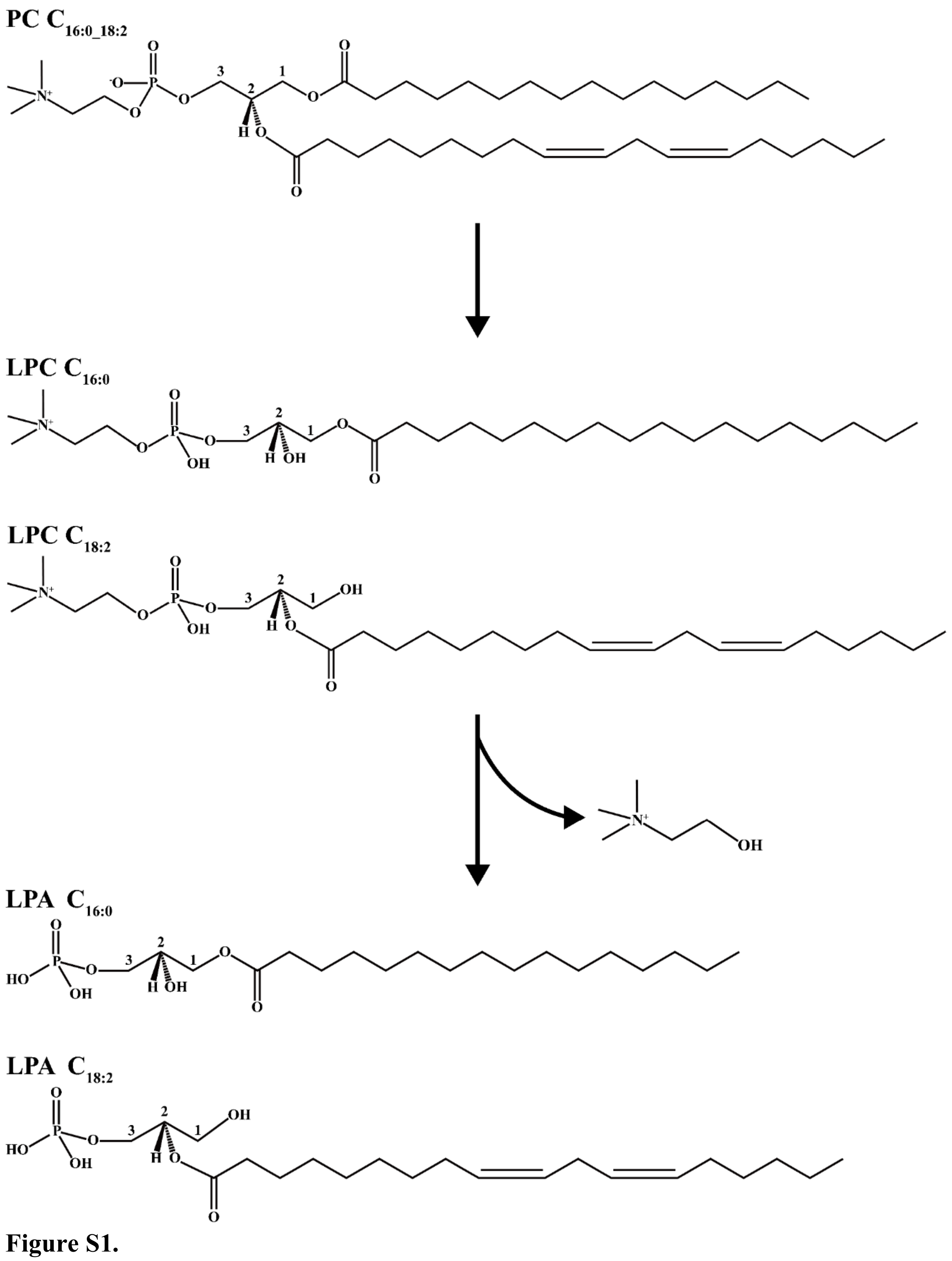

Figure S1.

A possible process for the lysophosphatidic acids (LPAs) formation from phospholipids such as phosphatidic acid (PA) or phosphatidylcholine (PC) in KRGM gintonin.
